# Does amitriptyline help for irritable bowel syndrome pain management?: An updated systematic review and meta-analysis

**DOI:** 10.1097/MD.0000000000046869

**Published:** 2026-01-02

**Authors:** Raghad Alhajaji, Manal Alfahmi, Eatedal Mohammed Alshareef, Alaa Mohammad Kadah Salim, Alshaymaa Khalid Mofareh, Sara Bandar Badirah, Ghosoon Mohammed Bafaraj, Sultan AlDhabei

**Affiliations:** a Makkah Branch of Ministry of Health, Saudi Ministry of Health, Makkah, Saudi Arabia; b Sleep Medicine Fellowship, King Saud University Medical City, Riyadh, Saudi Arabia; c Department of Public Health, King Abdullah Medical City, Makkah, Saudi Arabia; d Department of Neurology, King Abdullah Medical Complex, Jeddah, Saudi Arabia; e Department of Medicine, Batterjee Medical College, Jeddah, Saudi Arabia; f Department of Medicine, College of Medicine, Umm Al-Qura University, Makkah, Saudi Arabia; g Department of Diagnostic Radiology, King Faisal Specialist Hospital and Research Center, Jeddah, Saudi Arabia; h Department of Clinical Pharmacy, College of Pharmacy, Umm Al-Qura University, Makkah, Saudi Arabia; i Department of Medicine, College of Medicine, Najran University, Najran, Kingdom of Saudi Arabia.

**Keywords:** amitriptyline, IBS, irritable bowel syndrome, pain improvement, TCAs, tricyclic antidepressants

## Abstract

**Background:**

Irritable bowel syndrome (IBS) is one of the most common functional gastrointestinal disorders (FGIDs) worldwide. Amitriptyline is commonly prescribed for managing the symptoms of IBS as a second-line treatment. This study aimed to consolidate the existing evidence on the pain therapeutic efficacy of amitriptyline in adults with IBS.

**Methods:**

Following the PRISMA guidelines, we conducted an updated systematic review and meta-analysis of observational studies and randomized controlled trials, searching 4 databases (PubMed, Scopus, Web of Science, and the Cochrane Central Register of Controlled Trials) from inception until March 15, 2024.

**Results:**

The study included 15 studies involving 1497 patients with IBS, 12 of which were included in the meta-analysis. The main pooled rate of improvement significantly in amitriptyline was 66.61% (95% CI: 61.69–71.53%, fixed model); (*P* = .31; *I*^2^ = 5%). The pooled odd ratio was statistically significant in amitriptyline over the placebo 2.07 (95% CI: 1.48–2.91, fixed model); (*P* = .40; *I*^2^ = 1%). The findings conclude that amitriptyline is an effective treatment, precisely for improving IBS pain-related management.

**Conclusion:**

Amitriptyline significantly confers short-term improvements in abdominal pain and global IBS symptoms, but the certainty of evidence is limited by generally short follow-up, variable study quality, and incomplete reporting of safety and withdrawals. Longer, well-designed trials with standardized outcomes and systematic adverse-event monitoring are needed to clarify long-term effectiveness and tolerability.

## 1. Introduction

Irritable bowel syndrome (IBS) is defined under the Rome IV criteria as a functional gastrointestinal disorder characterized by chronic abdominal pain and altered bowel habits in the absence of detectable structural abnormalities.^[1]^ The key diagnostic requirement is recurrent abdominal pain occurring at least one day per week on average over the previous three months, with symptom onset at least 6 months before diagnosis.^[1]^ Furthermore, pain associated with 2 or more of the following features: improvement or worsening with defecation, changes in stool frequency (either increased or decreased), or changes in stool form (appearance/consistency as per the Bristol Stool Scale).^[1]^

The Rome IV criteria introduced several significant modifications from the previous Rome III version.^[1,2]^ These include increasing the required frequency of abdominal pain from at least three days per month to at least one day per week and eliminating “abdominal discomfort” as a qualifying symptom, focusing exclusively on pain.^[1,2]^ This refinement makes the Rome IV diagnosis more specific but potentially excludes milder cases that would have qualified under Rome III.^[2]^ The criteria also clarified that stool pattern abnormalities should be assessed only on days with abnormal bowel movements, rather than across all bowel movements.^[1]^

IBS is subtyped to include constipation-predominant (IBS-C), diarrhea-predominant (IBS-D), mixed (IBS-M), and unclassified (IBS-U).^[1]^ The Rome IV classification has been shown to identify a patient population with generally more severe symptoms and higher rates of comorbid psychological conditions like anxiety and depression compared to Rome III.^[3]^ However, some experts note this stricter definition may lead to underestimation of prevalence in community settings where milder symptoms are common.^[2]^

IBS prevalence varies globally; countries have different IBS prevalence rates, with the highest rate found in South America (21%) and the lowest in Southeast Asia (7.0%),^[4]^ reaching up to 10% globally.^[5]^ Females have higher prevalence (15.0% vs 11.0% for males).^[6]^ Research in Asia shows that fewer people meet the Rome IV criteria for IBS because more report bloating (70%) compared to pain (64.6%). The yearly rate of new cases fell from 134.79 to 89.35 per 10,000, a 33.7% drop, which is linked to stricter coding with the ICD-10 and the use of Rome IV guidelines.^[7]^ A meta-analysis of 57 studies (423,362 participants) found Rome III criteria yielded a 9.2% IBS prevalence (95% CI: 7.6–10.8), while Rome IV showed 3.8% (95% CI: 3.1–4.5). IBS-M was most common under Rome III (33.8% of cases), whereas IBS-D predominated under Rome IV (31.5%). Women had higher prevalence than men (12.0% vs 8.6%; odd ratio (OR) 1.46).^[5]^

The first line of IBS management typically entails providing dietary advice and implementing lifestyle modifications.^[8]^ The low FODMAP (fermentable oligosaccharides, disaccharides, monosaccharides, and polyols) diet is one such approach that restricts the intake of certain carbohydrates that are poorly absorbed and fermented in the gut, leading to symptoms such as bloating, gas, and diarrhea.^[8]^ First-line pharmacological therapy for IBS is for managing IBS-related pain, constipation, and diarrhea, such as anti-spasmodics, laxatives, soluble fibers, and antidiarrheal drugs.^[8]^ Despite their status as a second-line therapy, tricyclic antidepressants (TCAs) have yet to demonstrate a clear benefit.^[9]^

Previous studies show that amitriptyline is effective in relieving the pain-related symptoms of IBS.^[10]^ Amitriptyline is commonly prescribed due to its ability to modulate pain perception, affect gut motility, and impact neurotransmitters in the gut.^[11,12]^ Amitriptyline’s analgesic effects in IBS involve multiple mechanisms, primarily influencing central pain processing. It modulates both lateral and medial pain pathways, reducing visceral pain sensitivity and emotional distress by decreasing activation of the anterior cingulate cortex, a region essential for pain perception and emotional response.^[13–15]^ Additionally, amitriptyline increases central serotonin and norepinephrine levels, enhancing central pain modulation, and reduces locus coeruleus activity, thus dampening stress-induced pain responses.^[13–15]^ Although it possesses anticholinergic properties influencing gut motility, the primary therapeutic benefits stem from central nervous system effects rather than direct gastrointestinal actions.^[13–15]^

Despite the existence of earlier meta-analyses investigating the efficacy of amitriptyline for IBS-related pain, several gaps remain, including the emergence of new ROME IV criteria. This systematic review and meta-analysis aims to update the pooled results of the current evidence regarding the therapeutic effectiveness of amitriptyline in IBS patients, including a novel ATLANTIS randomized control trail.^[11]^

## 2. Methods

In reporting this systematic review and meta-analysis, the PRISMA statement guidelines were followed.^[16]^ All steps were conducted in strict accordance with the Cochrane Handbook of Systematic Reviews and Meta-analysis of Interventions (version 5.1.0).^[17]^

### 2.1. Eligibility criteria

Studies were eligible if they involved adults (≥18 years) diagnosed with IBS based on a clinical diagnosis or the Rome I, II, III, or IV criteria. The intervention had to be amitriptyline (any dose), compared to either a placebo or traditional therapy, with follow-up continuing until the study endpoint. The primary outcome was improvement rate among patients treated with amitriptyline and pain relief. Secondary outcomes were IBS symptoms severity score (IBS-SSS) mean changes and visual analogue scale (VAS score) mean changes of IBS-related pain with amitriptyline, changes in stools frequency and quality of life (QOL). Exclusions included studies that did not clearly differentiate IBS from other functional gastrointestinal disorders, those using antidepressants other than amitriptyline, those with unreliable for data extraction, studies lacking a full-text article, and non-English language publications.

### 2.2. Information sources and search strategy

A comprehensive search of 4 electronic databases (PubMed, Scopus, Web of Science, and the Cochrane Central Register of Controlled Trials) has been published between 1980 and 2024 (up to March 15, 2024) was conducted. Search terms included (“amitriptyline” OR “tricyclic antidepressants” OR “TCAs”) AND (“irritable bowel” OR “functional bowel syndrome” OR “irritable colon” OR “IBS”). Additionally, the references of the included studies were manually searched for any other potentially eligible studies.

### 2.3. Selection process

**Figure 1.**
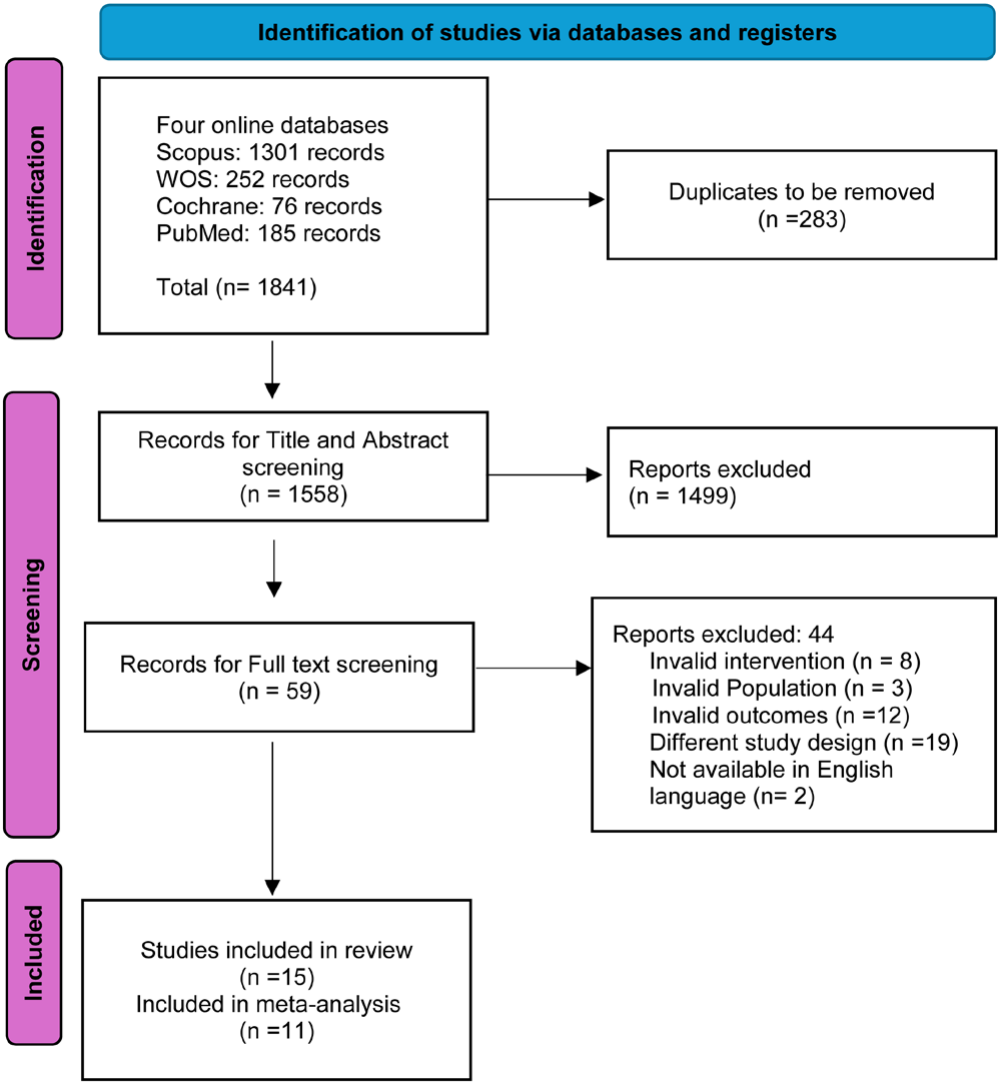
PRISMA flow diagram of studies’ screening and selection process.

After removing duplicates with EndNote (Clarivate Analytics, PA), 2 reviewers (RA, SB) independently screened the titles and abstracts on the Rayyan website, using the PICOS criteria on the uploaded titles and abstracts. Disagreements were resolved by a third reviewer. The second step involved screening the full-text articles of the identified abstracts for final eligibility for the meta-analysis, which was carried out by 2 independent reviewers (EA, MA), assisted by a third expert coauthor to resolve any conflicts. A uniform data extraction sheet held the extracted data. The extracted data included the characteristics of the included studies, the characteristics of the population of included studies, the risk of bias domains, and outcome measures. The PRISMA flow diagram (Fig. 1) illustrates the reasons for exclusion.

### 2.4. Assessing the risk of bias in the individual studies

The risk of bias of included studies has been assessed according to the NIH tool for single-arm observational studies, controlled intervention studies, and pre-and post-noncontrolled trials. For double-armed observational studies, the Newcastle–Ottawa scale was used. The authors’ judgments were categorized as “low risk,” “high risk,” or “unclear risk” of bias corresponding to the “poor,” “fair,” or “good” score they received. Table S1 (Supplemental Digital Content, https://links.lww.com/MD/R40).

### 2.5. Synthesis methods

Outcomes are either dichotomous data or continuous from prospectively designed studies. With the effect size used corresponding to the type of data, we calculated the pooled effect size for all outcomes according to the DerSimonian Laird meta-analysis model. This random effect model assumes the included studies represent a random sample from the population and assigns a slightly higher weight to small studies at the expense of larger studies. We choose between random and fixed models to make inconsistent or controversial estimates. R.4.4.1 software with meta-backup software was utilized.

### 2.6. Assessment of heterogeneity

Statistical heterogeneity among studies was evaluated using the chi-square test (Cochran *Q* test). Then, the χ^2^ statistic, Cochran *Q*, was used to calculate the *I*^2^ according to the equation: $I^{2}=\left(\frac{Q-df}{Q}\right)\times 100\%.$ A χ^2^ *P* value of <.1 was considered as significant heterogeneity. *I*^2^ values ≥50% were indicative of high heterogeneity.

### 2.7. Certainty assessment

To test the robustness of the evidence, we conducted a certainty assessment through sensitivity analysis (also called leave-one-out meta-analysis). For every outcome in the meta-analysis, we ran sensitivity analysis in multiple scenarios, excluding one study in each scenario to ensure that the overall effect size was not solely dependent on any single study. Figures S1–S3 (Supplemental Digital Content, https://links.lww.com/MD/R40).

## 3. Results

### 3.1. Literature search results

Our literature search process retrieved 1841 records. Following the title and abstract screening, 189 articles were eligible for full-text screening. Of them, 15 studies were included in the systematic review, while 12 studies were included in the meta-analysis. The references of the included studies were manually searched, and no further articles were included. The PRISMA flow diagram of the study selection process is shown in Figure 1.

### 3.2. Characteristics of the included studies

Fifteen studies were included in the systematic review, with a total of 1497 patients with IBS. In double-arm studies, patients were assigned to receive either amitriptyline or a placebo. A summary of the included studies is provided in Tables 1 and 2. Overall, the risk of bias in the included studies ranged from moderate to low risk of bias according to the NIH tool and Newcastle–Ottawa scale checklists according to different study designs, as shown in Table S1 (Supplemental Digital Content, https://links.lww.com/MD/R40). Methodological quality was high across the evidence base. Of 15 studies, 12 (80%) were rated “Good” and 3 (20%) “Fair,” with none “Poor.” The comparative observational study was good (NOS 8/9); all 6 before–after studies without controls were good (scores 8–10; median 9); and among 7 controlled intervention studies, five were good (9–10) and 2 fair (8). Only the single-arm observational study was fair (score 7). These ratings indicate generally strong rigor and a low overall risk of bias.

**Table 1:** Summary table of included studies in the systematic review and meta-analysis.

Study ID	Country	Study design	Sample size	Interventional group	Control group	Follow-up	Main outcomes	Included in MA	Etiology	Overall quality
Bahar^[18]^ (2008)	USA	RCT	33	Amitriptyline (10–30 mg od)	Placebo	13 wk	IBS-QOL questionnaire, IBS-associated pain and diarrhea percentage change, change in days with pain, and change in frequency of stool.	Yes	Fulfilled criteria	Good
Clouse^[19]^ (1994)	USA	Observational	150	Amitriptyline (50 mg od)	NA	1.5 yr	Symptoms improvement.	Yes	Fulfilled criteria	Fair
Ford^[11]^ (2023)	UK	RCT	463	Amitriptyline (10–30 mg od)	Placebo	6 mo	Symptoms improvement (IBS-SSS score), PHQ-12, HADS, and adverse events.	Yes	Fulfilled criteria	Good
Khosravi^[20]^ (2022)	Iran	Observational	100	Amitriptyline (25 mg od)	Mesalazine	4 wk	Symptoms improvement, IBS-associated pain improvement (VAS), and stool frequency.	Yes	Fulfilled criteria	Good
Li^[21]^ (2019)	China	RCT	139	Amitriptyline (25 mg od)	L. acidophilus	1 yr	Symptoms improvement (IBS-SSS) and change in days with pain.	Yes	Fulfilled criteria	Good
Mertz^[22]^ (1998)	USA	RCT	7	Amitriptyline (50 mg od)	Placebo	11 wk	Symptoms improvement and IBS-associated pain improvement (VAS).	Yes	Fulfilled criteria	Fair
Mishra^[23]^ (2014)	India	RCT	108	Amitriptyline (25 mg od)	Mebeverine	2 wk	GSRS and HADs scale.	No	No valid outcomes for MA	Good
Morgan^[14]^ (2005)	USA	RCT	22	Amitriptyline (50 mg od)	Placebo	1 mo	Symptoms improvement and IBS-associated pain change (VAS score).	Yes	Fulfilled criteria	Good
Otaka^[24]^ (2005)	Japan	RCT	81	Amitriptyline (30 mg od)	NA	4 wk	Symptoms improvement.	Yes	Fulfilled criteria	Good
Poitras^[25]^ (2002)	Canada	RCT	39	Amitriptyline (10 mg od for 2 wks, 25 mg od for 4 wks)	Psychotherapy	10 wk	Gastrointestinal index, QOL index.	Yes	Fulfilled criteria	Good
Rajagopalan^[26]^ (1998)	India	RCT	40	Amitriptyline (25 mg od for 1 wk, 50 mg od for 1 wk, 75 mg od thereafter)	Placebo	12 wk	Symptoms improvement, change in days with pain, and stool frequency per day.	Yes	Fulfilled criteria	Fair
Sohn^[27]^ (2012)	Korea	RCT	228	Amitriptyline (10 mg od)	Tianeptine	4 wk	Symptoms improvement, IBS-associated pain improvement (VAS), EQ-5D score, adherence, stool frequency, and adverse events.	Yes	Fulfilled criteria	Good
Steinhart^[28]^ (1981)	USA	RCT	1.n4	Amitriptyline (50 mg od)	Placebo	4 wk	Response to amitriptyline as a function of MMPI.	No	No valid outcomes for MA	Fair
Thoua^[12]^ (2008)	UK	RCT	19	Amitriptyline (25–50 mg od)	NA	3 mo	Symptoms improvement (IBS-SSS).	Yes	Fulfilled criteria	Good
Vahedi^[29]^ (2008)	Iran	RCT	54	Amitriptyline (10 mg od)	Placebo	2 mo	Symptoms improvement and adverse events.	Yes	Fulfilled criteria	Good

EQ-5D score = European quality of life five dimensions questionnaire, GSRS = gastrointestinal symptom rating scale, HADS = hospital anxiety and depression scale, IBS = irritable bowel syndrome, IBS-SSS = IBS symptoms severity score, MMPI = Minnesota Multiphasic Personality Inventory, NA = not available, PHQ-12 = patient health quality questionnaire-12 questions, QOL = quality of life, RCT = randomized controlled trials, USA = United States of America, VAS scale = visual analogue scale, wk = week.

**Table 2:** Baseline table of included studies in systematic review and meta-analysis.

Study ID	Age* (yr)	Gender	Duration of disease	IBS diagnostic criteria†	IBS subtype‡
Bahar^[18]^ (2008)	Mean: (15.3)	9 M, 24 F	2002–2005	ROME II	NA
Clouse^[19]^ (1994)	Median (range): 47 (16–79)	57 M, 93 F	5 yr	Clinically	NA
Ford^[11]^ (2023)	Mean (SD): 48.5 (16.1)	148 M, 315 F	Median 10 years (IQR 4–21)	Rome IV	IBS-D, C, M, U
Khosravi^[20]^ (2022)	Mean (SD): 31.08 (6.38)	40 M, 60 F	NA	ROME III	IBS-D
Li^[21]^ (2019)	Mean (SD): 41.32 (9.82)	88 M, 82 F	2015–2016	Clinically	IBS-D refractory
Mertz^[22]^ (1998)	Mean (43.6)	NA	NA	ROME (unspecified)	NA
Mishra^[23]^ (2014)	18 yr or older	NA	6 mo	ROME II	NA
Morgan^[14]^ (2005)	Mean (range): 39 (24–57)	NA	NA	ROME II	IBS-D, C, U
Otaka^[24]^ (2005)	Mean (SD): 71.5 (7.1)	33 M, 48 F	NA	ROME II	NA
Poitras^[25]^ (2002)	Mean (range): 41.1 (35–64)	5 M, 7 F	NA	Rome I	NA
Rajagopalan^[26]^ (1998)	Mean: 35.1 yr	11 M,11 F§	NA	ROME (unspecified)	NA
Sohn^[27]^ (2012)	Mean (SD): 51 (13)	N/A	N/A	Rome III	IBS-D
Steinhart^[28]^ (1981)	Mean: (41)	3 M, 11 F	5.07 yr	Clinically and lab	NA
Thoua^[12]^ (2008)	Median: (32)	3 M, 16 F	NA	ROME II	IBS-C, D
Vahedi^[29]^ (2008)	Mean: (36)	29 M, 21 F	NA	ROME II	IBS-D

F = female, IBS = irritable bowel syndrome, M = male, NA = not available, ROMI = rating of medication influences, SD = standard deviation.

* Age of amitriptyline group.

† Clinically, laboratory, both, ROME criteria I, II, III.

‡ IBS-D: Irritable bowel syndrome with predominant diarrhea/ IBS-C: Irritable bowel syndrome with predominant constipation/ IBS-M: Irritable bowel syndrome mixed bowel habits/ IBS-U: Irritable bowel syndrome unclassified.^[38]^

§ 18 patients dropped out.

In our main analysis, we included nine studies. We did not assess publication bias (funnel plots or Egger test) because these methods are underpowered when the number of studies is small (fewer than 10).

### 3.3. Overall IBS related symptom improvement

**Figure 2.**
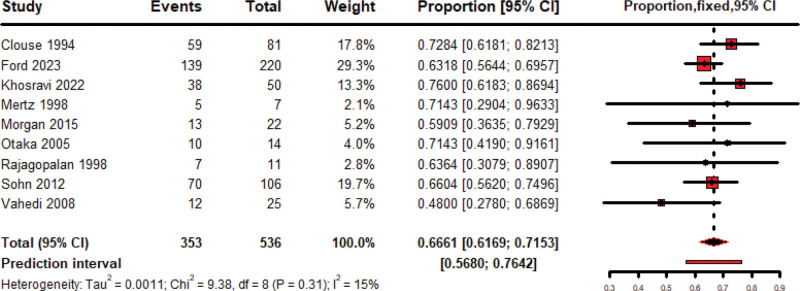
Forest plot of pooled meta-analysis of IBS improvement rate by amitriptyline. IBS = irritable bowel syndrome.

**Figure 3.**
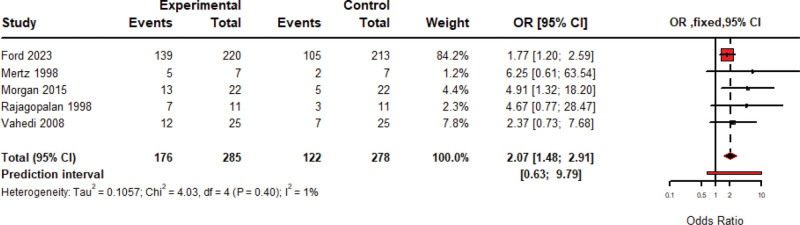
Forest plot of pooled meta-analysis of IBS improvement rate by amitriptyline compared to a placebo. IBS = irritable bowel syndrome.

**Figure 4.**
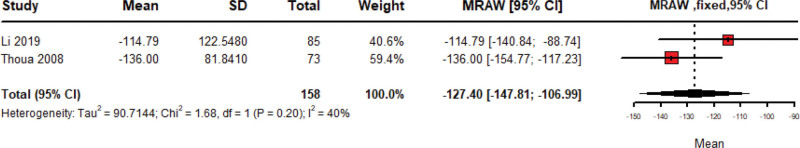
Forest plot of pooled meta-analysis of IBS improvement by amitriptyline using IBSS-SSS rating. IBS = irritable bowel syndrome, IBS-SSS = IBS symptoms severity score.

The pooled rate of improvement of 536 patients treated by amitriptyline over nine studies is 66.61% (95% CI: 61.69–71.53, Fixed model), included studies were homogeneous (*P* = .31; *I*^2^ = 15%) as shown in Figure 2. When comparing the improvement over 285 patients treated by Amitriptyline with 278 others treated by placebo, over five included studies, the pooled OR favored Amitriptyline over placebo by 2.07 (95% CI: 1.48–2.91, Fixed model) with non-significant heterogeneity detected among included studies (*P* = .40; *I*^2^ = 1%) as shown in Figure 3. IBS-SSS score mean changes after Amitriptyline treatment have been pooled, giving a pooled mean change of −127.40 (95% CI: −147.81 to −106.99, Fixed Model), with no heterogeneity among included studies (*P* = .20; *I*^2^ = 40%) as shown in Figure 4.

### 3.4. IBS-related pain change

**Figure 5.**
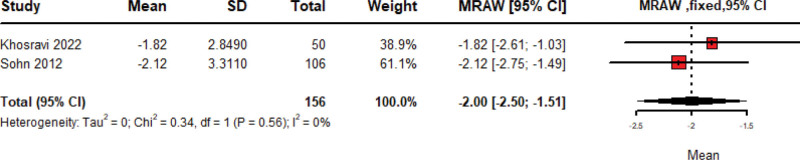
Forest plot of pooled meta-analysis of VAS score mean change of IBS-related pain with amitriptyline. IBS = irritable bowel syndrome, VAS = visual analogue scale.

The pooled VAS score means change of IBS-related pain in 156 patients treated with Amitriptyline is −2.00 (95% CI: −2.50 to −1.51, Fixed model) with no heterogeneity among included studies (*P* = .56; *I*^2^ = 0%) as shown in Figure 5. While the mean change of days reported with pain after amitriptyline treatment, 107 patients over 3 studies, is −10.14 (95% CI: −23.03–2.76, Random model) with statistically significant heterogeneity among included studies (*P* < .01; *I*^2^ = 92%) as shown in Figure S1 (Supplemental Digital Content, https://links.lww.com/MD/R40). Leave-one-out sensitivity analysis was done, and heterogeneity was resolved by the removal of Li 2019 as shown in Figure S1 (Supplemental Digital Content, https://links.lww.com/MD/R40).

### 3.5. Stool frequency

**Figure 6.**
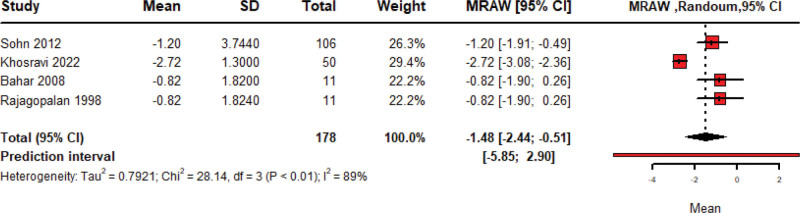
Forest plot of pooled meta-analysis of change in stool frequency after amitriptyline.

The pooled mean change of stool frequency in 178 patients treated with Amitriptyline is −1.48 (95% CI: −2.44 to −0.51, random model) with significant heterogeneity among included studies (*P* < .01; *I*^2^ = 89%) as shown in Figure 6. Leave-one-out sensitivity analysis was done, and heterogeneity was resolved by the removal of Khosravi 2022 as shown in Figure S2 (Supplemental Digital Content, https://links.lww.com/MD/R40).

### 3.6. Quality of life

**Figure 7.**
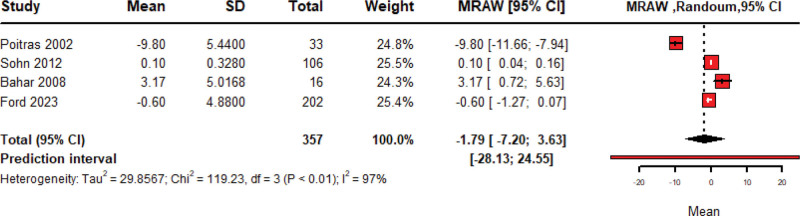
Forest plot of pooled meta-analysis of change in quality of life after amitriptyline.

The pooled mean change in QOL over 357patients treated with Amitriptyline is −1.79 (95% CI: −7.20 to −3.63, random model) with significant heterogeneity among included studies (*P* < .01; *I*^2^ = 97%) as shown in Figure 7. Leave-one-out sensitivity analysis was done, and heterogeneity cannot be resolved by the removal of any of the included studies as shown in Figure S3 (Supplemental Digital Content, https://links.lww.com/MD/R40).

## 4. Discussion

After screening 1841 records, we included 15 studies, totaling 1497 IBS patients, of which we used 12 in the meta-analysis. Amitriptyline significantly improved IBS symptoms in 536 patients, producing a 66.61% improvement rate (95% CI: 61.69–71.53, *I*²=15%, *P* = .31). Comparing 285 amitriptyline recipients with 278 placebo recipients revealed a pooled odds ratio of 2.07 (95% CI: 1.48–2.91, *I*^2^=1%, *P* = .40). IBS-SSS mean change was–127.40 (95% CI: –147.81 to –106.99), and amitriptyline reduced VAS pain scores by–2.00 (95% CI: –2.50 to –1.51).

Findings demonstrate further efficacy for managing IBS-related symptoms, and many published results showed that treatment was successful and provide statistically significant evidence that amitriptyline is effective in decreasing stool frequency. Rahimi et al reported that TCA therapy without specifically investigating amitriptyline, significantly improved IBS symptoms (RR: 1.93, 95% CI: 1.44–2.6) and reduced abdominal pain by 44.15 points (95% CI: −53.27 to −35.04), both with *P* < .0001,^[30]^ which similar to our findings.

Compared to previous meta-analyses, it includes the highest number of amitriptyline-focused articles, such as the ATLANTIS randomized clinical trial by Alexander C. Ford et al, the largest study on amitriptyline as a second-line treatment, involving 463 participants from 55 general practice clinics in England. Moreover, Amitriptyline (10–30 mg) outperformed placebo in IBS-SSS at 6 months (−27.0 [−46.9 to − 7.10], *P* = .0079), with 20% versus 26% discontinuation.^[11]^ Likewise, titrated low-dose amitriptyline was more effective in improving IBS-related symptoms than the placebo and was safe and well tolerated when titrated according to symptom response and the adverse effects of 13% (amitriptyline) versus 9% (placebo).^[11]^ Ford et al conducted a meta-analysis, which supports the finding that TCAs are more effective than placebos in reducing persistent IBS symptoms.^[31]^

Other options to manage pain in IBS, including mesalazine, an anti-inflammatory drug, may reduce both pain and diarrhea by lowering mast cell activity in the gut,^[20]^ and antispasmodics like hyoscine and dicyclomine relax the muscles in the gut to relieve pain from spasms, while peppermint oil offers natural relief with its antispasmodic properties.^[15]^ Likewise, nortriptyline and desipramine reduce chronic pain by modulating pain signals in the brain.^[15]^ When it comes to comparing TCA with SSRI’s meta-analysis, Xie meta-analysis results of TCA risk ratio (RR) favored TCAs significantly (RR = 1.36, 95% CI: 1.07–1.71, *P* = .01), with low heterogeneity (*I*^2^ = 21%). Whereas SSRIs subgroup did not show a significant benefit (RR = 1.38, 95% CI: 0.83–2.28, *P* = .21) and (*I^2^* = 75%). Furthermore, both TCA and SSRI’s shows no statistically significant difference in QoL improvement compared to control (mean difference = 0.39; 95% CI: −3.19 to 3.97, *P* = .83).^[32]^ The overall heterogeneity was substantial (*I*^2^ = 70%).^[32]^ Analogously, our results were −1.79 (95% CI: −7.20 to 3.63) of improving QoL.

Across the studies reviewed, amitriptyline was commonly associated with anticholinergic side effects, including dry mouth, drowsiness, constipation, dizziness, and urinary retention.^[11,14,15,21,31]^ Although, these adverse effects were generally mild and transient, typically resolving within one to 2 weeks in most patients.^[21,29]^ Consequently, in a large multicenter trial by Ford et al (2023), 13% of participants in the amitriptyline group discontinued treatment due to adverse events, compared to 9% in the placebo group, with sedation and dry mouth being the most frequently reported issues.^[11]^ In a double-blind, placebo-controlled trial involving adolescents with IBS, no participants discontinued treatment after initiation, indicating good short-term tolerability.^[18]^ Electrocardiograms (ECGs) were performed as a safety measure, and no cardiac abnormalities were observed.^[18]^ Similarly, in the study by Zhou et al (2021), which primarily investigated pharmacogenetic predictors of treatment response, the safety profile of amitriptyline was not extensively explored.^[33]^ However, one patient discontinued treatment due to “unbearable sleepiness” attributed to the drug.

In a head-to-head comparison of tianeptine and amitriptyline, Sohn et al (2012) found that amitriptyline was associated with a significantly higher incidence of adverse effects, particularly dry mouth (20% vs 7%) and constipation (6% vs 1%). Notably, no serious adverse events occurred in either treatment arm.^[27]^

An essential consideration is that amitriptyline is often used as a chronic treatment, which underscores the importance of understanding its long-term safety, tolerability, and the durability of its therapeutic’s effects. While the follow-up in the included studies is <1 year. The lack of long-term follow-up in clinical trials evaluating amitriptyline for (IBS) presents significant limitations in assessing sustained therapeutic benefits. In Ford’s trial, the follow-up was curtailed due to COVID-19 disruptions, resulting in a reduced sample size and the loss of randomization.^[11]^ Similarly, another study (Morgan et al, 2005) did not acknowledge the short one-month follow-up as a limitation, despite recognizing that the study was underpowered to detect significant changes in pain sensitivity and clinical outcomes.^[14]^

Furthermore, in the study by Wei-Dong Li (2018), the inclusion of a 1-year follow-up period did not address the absence of follow-up beyond this time frame.^[21]^ While the study focused on the immediate effects of low-dose amitriptyline on refractory diarrhea-predominant IBS, the reasons for not extending the follow-up period beyond 1 year were not discussed.^[21]^

Shorter studies, such as Bahar et al (2008) and Zhou et al (2021), support short-term efficacy but are limited by brief durations and small sample sizes. Although most included studies assess short-term outcomes (typically 4 to 12 weeks), longer-term data remain limited.^[18,33]^ Notably, Clouse et al (1994) provide important longitudinal evidence on antidepressant use for IBS, reporting a mean follow-up duration of 1.5 years.^[19]^ Among patients who achieved symptom remission, 77% remained symptom-free during continued treatment. Overall, 89% reported symptom improvement, and 61% achieved complete remission – particularly those with pain-predominant IBS.^[19]^ Furthermore, 46% of patients who switched to an alternative agent following initial non-response ultimately achieved remission, highlighting the potential utility of sequential antidepressant trials in treatment-resistant cases.^[19]^

IBS is often influenced by underlying psychological factors. Both anxiety and depression can exacerbate gastrointestinal symptoms and are responsive to antidepressant therapy. This raises an important question in IBS pharmacotherapy: are the observed improvements with amitriptyline the result of direct neuromodulators effects on the gastrointestinal tract, or are they secondary to improved psychological well-being? Given that a substantial proportion of IBS patients experience psychological distress, the failure to assess or adjust for these variables introduces a risk of confounding. Among the studies reviewed, approaches to addressing psychological factors varied considerably, with important implications for interpreting treatment outcomes.

Many trials, the ATLANTIS trial and Thoua et al (2008) monitored psychological status using validated instruments such as the hospital anxiety and depression scale.^[11,12]^ Both reported that IBS symptom improvement occurred independently of changes in anxiety or depression, suggesting a direct peripheral or neuro-gastroenteric mechanism of action for amitriptyline. In contrast, several other studies, including those by Clouse et al (1994), Bahar et al (2008), and Zhou et al (2021), did not assess baseline psychological status or failed to report on changes in mood during treatment.^[18,19,33]^ For instance, Bahar et al reasoned that the low doses used (10–30 mg/day), well below typical antidepressant levels, were acting as neuromodulators rather than as mood-elevating agents.^[18]^ However, without psychological data, these studies cannot rule out the possibility that observed improvements were mediated, at least in part, by enhancements in psychological well-being.

This study advances previous literature by providing updated evidence that reflects the current understanding of IBS management. It specifically differentiates the effects of amitriptyline from other pharmacological interventions. There are several key strengths in this study. First, many previous reviews were limited by the availability of newer randomized controlled trials (RCTs), restricting their scope to older diagnostic criteria (e.g., Rome III or earlier) and potentially missing recent evidence. Second, earlier syntheses often combined data from various antidepressants without clearly isolating amitriptyline’s specific impact on pain, or they did not conduct sensitivity analyses to assess the robustness of pooled outcomes. Finally, our study includes both single-arm and double-arm trials that focus on amitriptyline usage.

To address these gabs in previous studies, our meta-analysis encompasses studies published up to 2024, incorporating more recent RCTs and expanded patient populations that reflect real-world clinical diversity. By filling these gaps, our analysis provides an updated and clinically relevant assessment of amitriptyline’s role as a second-line therapy in the management of IBS-related pain.

Despite statistical significance, this study like others has limitations. These include the variation in amitriptyline doses, formulations, withdrawal rate due to side effects, improvement with each subgrouping of IBS and treatment durations among the included studies. Additionally, there is a lack of modification in the diagnostic criteria, introducing observational study bias. The limited number of studies may not have had adequate power to detect significant differences in evaluating both VAS scores, IBS-SSS scores, adherence, and the effect on QOL. Loss of long-term safety data also remains a concern. Safety outcomes were not defined well across the included studies, while withdrawal rates due to adverse events were captured in a variable manner. This inconsistency limits the clinical applicability of the findings and precludes robust pooling of harm data. The lack of standardization hinders our ability to draw meaningful conclusions regarding the overall safety profile of the interventions being reviewed. Further efforts are required to establish clear definitions of safety and long-term follow-up according to reporting guidelines to improve the reliability of future research in this area.

### 4.1. Conclusion

This study is the latest and most relevant, supports amitriptyline medication in IBS patients, particularly in patients with pain-predominant symptoms. Nevertheless, the lack of prospective long-term controlled trials remains a key gap in the evidence base. Further research is needed to establish the long-term safety, efficacy, and optimal duration of amitriptyline therapy in the management of IBS.

## Acknowledgments

We would like to express our gratitude to Dr Rehab Diab for her invaluable guidance in analysis process.

## Author contributions

**Conceptualization:** Raghad Alhajaji.

**Data curation:** Raghad Alhajaji, Manal Alfahmi, Alaa Mohammad Kadah Salim, Alshaymaa Khalid Mofareh.

**Formal analysis:** Raghad Alhajaji.

**Funding acquisition:** Raghad Alhajaji.

**Investigation:** Raghad Alhajaji, Manal Alfahmi, Eatedal Mohammed Alshareef, Alaa Mohammad Kadah Salim, Sara Bandar Badirah, Ghosoon Mohammed Bafaraj.

**Methodology:** Raghad Alhajaji, Manal Alfahmi, Eatedal Mohammed Alshareef, Sara Bandar Badirah, Ghosoon Mohammed Bafaraj, Sultan AlDhabei.

**Project administration:** Eatedal Mohammed Alshareef, Alshaymaa Khalid Mofareh.

**Resources:** Manal Alfahmi, Eatedal Mohammed Alshareef, Alshaymaa Khalid Mofareh.

**Software:** Raghad Alhajaji.

**Supervision:** Raghad Alhajaji, Manal Alfahmi, Sultan AlDhabei.

**Validation:** Raghad Alhajaji, Manal Alfahmi, Eatedal Mohammed Alshareef, Alaa Mohammad Kadah Salim, Alshaymaa Khalid Mofareh, Ghosoon Mohammed Bafaraj.

**Visualization:** Raghad Alhajaji, Alaa Mohammad Kadah Salim, Sara Bandar Badirah.

**Writing – original draft:** Raghad Alhajaji, Manal Alfahmi, Eatedal Mohammed Alshareef, Alaa Mohammad Kadah Salim, Alshaymaa Khalid Mofareh, Sara Bandar Badirah, Ghosoon Mohammed Bafaraj, Sultan AlDhabei.

**Writing – review & editing:** Raghad Alhajaji, Manal Alfahmi, Eatedal Mohammed Alshareef, Alaa Mohammad Kadah Salim, Alshaymaa Khalid Mofareh, Sara Bandar Badirah, Ghosoon Mohammed Bafaraj, Sultan AlDhabei.

## Supplementary Material


