# Supplementary material for: Does amitriptyline help for irritable bowel syndrome pain management?: An updated systematic review and meta-analysis
Source: Medicine (Baltimore). 2026 Jan 2;105(1):e46869. doi: 10.1097/MD.0000000000046869 (PMC12778161; doi:10.1097/MD.0000000000046869)
Supplement: Supplementary file 1 [file medi-105-e46869-s001.docx]

**Supplementary figure S1: Leave-one-out sensitivity analysis of change in days with pain.**

**
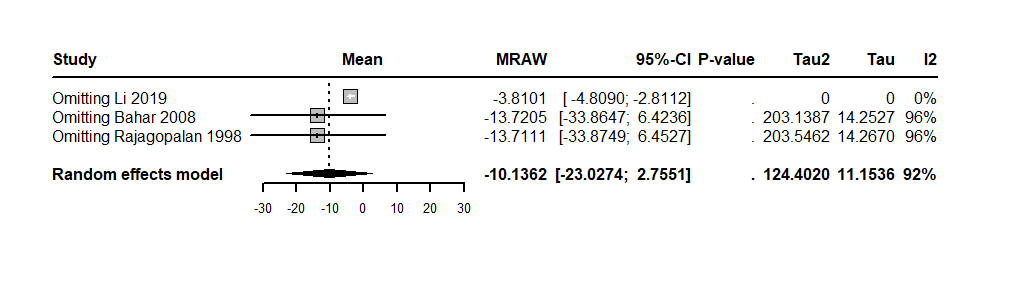
**

**Supplementary Figure S2 : Leave-one-out sensitivity analysis of change in stool frequency.**

**
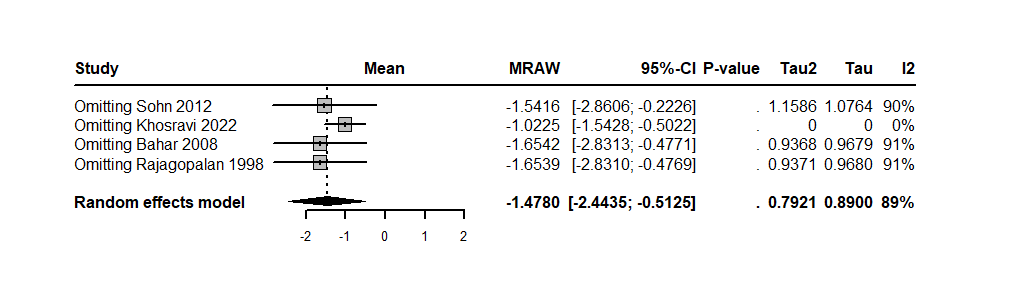
**

**Supplementary Figure S3: Leave-one-out sensitivity analysis of change in Quality of life.**

**
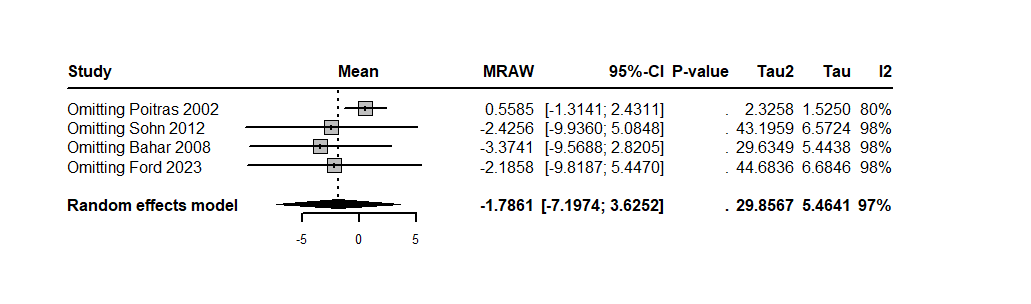
**

**Supplementary Table S1: Quality assessment of the included studies.**

| **Study ID** | **Quality assessment for Single-arm observational studies according to the NIH tool** | | | | | | | | | | | | | | | |
| --- | --- | --- | --- | --- | --- | --- | --- | --- | --- | --- | --- | --- | --- | --- | --- | --- |
|  | **Q1** | **Q2** | **Q3** | **Q4** | **Q5** | **Q6** | **Q7** | **Q8** | **Q9** | **Q10** | **Q11** | **Q12** | **Q13** | **Q14** | **Overall Score** | **Overall quality** |
| Clouse 1994 | Y | Y | Y | Y | CD | Y | N | N | Y | N | Y | N | N | CD | 7 | Fair |
| **Study ID** | **Quality assessment for Comparative observational studies according to NOS** | | | | | | | | | | | | | | | |
|  | **Selection** | | | | | | | | **Compatibility** | **Outcome** | | | | | | |
|  | **Q1** | | **Q2** | | **Q3** | | **Q4** | | **Q5** | **Q6** | | **Q7** | **Q8** | | **Overall Score** | **Overall quality** |
| Khosravi 2022 | * | | * | | * | | * | | ** | * | |  | * | | 8 | Good |
| **Study ID** | **Quality assessment for Before-After (Pre-Post) Studies with No Control Group** | | | | | | | | | | | | | | | |
|  | **Q1** | **Q2** | **Q3** | **Q4** | **Q5** | **Q6** | **Q7** | **Q8** | **Q9** | **Q10** | **Q11** | | **Q12** | | **Overall Score** | **Overall quality** |
| Otaka 2005 | Y | Y | Y | Y | CD | Y | Y | N | Y | N | Y | | CD | | 8 | Good |
| Mishra 2014 | Y | Y | Y | Y | Y | Y | Y | N | Y | N | Y | | CD | | 9 | Good |
| Poitras 2002 | Y | Y | Y | N | Y | Y | Y | N | Y | Y | N | | N | | 8 | Good |
| Sohn 2012 | Y | Y | Y | Y | Y | Y | Y | N | Y | N | Y | | CD | | 9 | Good |
| Thoua 2008 | Y | Y | Y | Y | Y | Y | Y | N | Y | Y | Y | | N | | 10 | Good |
| Li 2019 | Y | Y | Y | Y | CD | Y | Y | N | Y | Y | Y | | CD | | 10 | Good |
| **Study ID** | **Quality assessment for Controlled Intervention Studies** | | | | | | | | | | | | | | | |
|  | **Q1** | **Q2** | **Q3** | **Q4** | **Q5** | **Q6** | **Q7** | **Q8** | **Q9** | **Q10** | **Q11** | **Q12** | **Q13** | **Q14** | **Overall Score** | **Overall quality** |
| Bahar 2008 | Y | Y | Y | Y | CD | Y | Y | N | Y | N | Y | N | Y | CD | 9 | Good |
| Morgan 2005 | Y | Y | Y | Y | Y | Y | Y | N | N | Y | Y | CD | Y | N | 10 | Good |
| Ford 2023 | Y | Y | Y | Y | Y | Y | Y | N | Y | N | Y | N | N | N | 9 | Good |
| Vahedi 2008 | Y | Y | Y | Y | Y | Y | Y | N | N | Y | Y | CD | Y | N | 10 | Good |
| Rajagopalan 1998 | Y | Y | Y | N | CD | Y | Y | N | Y | Y | Y | CD | CD | N | 8 | Fair |
| Mertz 1998 | Y | Y | Y | N | CD | Y | Y | N | Y | N | N | Y | CD | CD | 8 | Fair |
| Steinhart 1981 | Y | Y | Y | N | CD | Y | Y | N | Y | N | N | N | CD | CD | 7 | Fair |

**NIH tool for single-arm observational studies**: Q1: Was the study question or objective clearly stated? Q2: Was the study population clearly specified and defined? Q3: Was the participation rate of eligible persons at least 50%? Q4: Were all the subjects selected or recruited from the same or similar populations (including the same time period)? Were inclusion and exclusion criteria for being in the study prespecified and applied uniformly to all participants?, Q5: Was a sample size justification, power description, or variance and effect estimates provided?, Q6: For the analyses in this paper, was the exposure(s) of interest measured prior to the outcome(s) being measured?, Q7: Was the timeframe sufficient so that one could reasonably expect to see an association between exposure and outcome if it existed?, Q8: For exposures that can vary in amount or level, did the study examine different levels of the exposure as related to the outcome (e.g., categories of exposure, or exposure measured as a continuous variable)?, Q9: Were the exposure measures (independent variables) clearly defined, valid, reliable, and implemented consistently across all study participants?, Q10: Was the exposure(s) assessed more than once over time?, Q11: Were the outcome measures (dependent variables) clearly defined, valid, reliable, and implemented consistently across all study participants?, Q12: Were the outcome assessors blinded to the exposure status of participants?, Q13: Was loss to follow-up after baseline 20% or less?, Q14: Were key potential confounding variables measured and adjusted statistically for their impact on the relationship between exposure(s) and outcome(s)?. **NOS tool for Comparative Observational studies:** Three domains: Selection, Compatibility and Outcome domains. Q1: Representativeness of the sample, Q2: Selection of the non-exposed cohort, Q3: Ascertainment of Exposure, Q4: Demonstration that that outcome of interest was not present at the start of the study, Q5: comparability Of cohorts based on design or analysis controlled for confounders, Q6: Assessment of outcome, Q7: was follow up long enough for outcomes to occur, Q8: Adequacy of Follow-up. **NIH tool for Controlled intervention studies:** Q1:Adequate randomization method? Q2:Treatment allocation concealed. Q3: Blinding of participants and providers?, Q4: Blinding of outcome assessors?, Q5: Are groups similar at baseline?, Q6: Endpoint drop-out rate ≤ 20%?, Q7: Differential drop-out rate ≤ 15%?, Q8: High adherence to intervention protocols?, Q9: Avoidance of other interventions?, Q10: Valid and reliable outcome measures?, Q11: Sample size adequate for 80% power?, Q12: Prespecified outcomes or subgroups? Q13: Intention-to-treat analysis used? **NIH tool for Before-After (Pre-Post) Studies With No Control Group:** Q1: Clear study question/objective?, Q2: Prespecified eligibility criteria?, Q3: Representative study population?, Q4: Enrollment of eligible participants?, Q5: Sufficient sample size?,Q6: Clear and consistent intervention description?, Q7: Prespecified, valid outcome measures?,Q8: Blinded outcome assessors?, Q9: Loss to follow-up ≤ 20%, accounted for?,Q10: Statistical tests for pre-post changes?, Q11: Interrupted time-series design?,Q12: The analysis accounted for group-level intervention. **Y**, yes; **N**, no; **NA**, not applicable; **CD**: cannot be determined.
